# Investigation on the Factors Affecting the Exhaust Degradation Performance of Porous Pavement Mixtures with Nano-TiO_2_ Photocatalysts

**DOI:** 10.3390/ma18051139

**Published:** 2025-03-03

**Authors:** Wenke Yan, Congwei Bi, Chuan Lu, Jikai Fu, Mulian Zheng, Qiang Ding, Jiasheng Liu

**Affiliations:** 1 Key Laboratory for Special Area Highway Engineering of Ministry of Education, Chang’an University, Xi’an 710064, China; 2022221260@chd.edu.cn (W.Y.); clu@chd.edu.cn (C.L.); 2 Shandong Hi-Speed Infrastructure Construction Co., Ltd., Jinan 250000, China; 17660106996@163.com (C.B.); 17753355660@163.com (J.F.); 13739821837@163.com (Q.D.); 3 Zibovocational Institute, Zibo 255300, China; 4 Shaanxi Zhouzhi County Transportation Bureau, Xi’an 710064, China; 13774936743@163.com

**Keywords:** photocatalytic pavement, porous asphalt mixtures, porous cement concrete, exhaust degradation

## Abstract

The substantial voids of porous pavement materials permit light and exhaust pollutants to infiltrate to a considerable depth. Consequently, utilizing porous mixtures as carriers for photocatalytic materials enables greater exposure to an environment conducive to the exhaust degradation reaction. This study employed porous asphalt mixtures and porous cement concrete as carriers for photocatalytic pavements. Various amounts of TiO_2_ were incorporated as photocatalysts to produce eco-friendly pavement materials with exhaust degradation capability. Based on a self-developed apparatus and methodology, its exhaust degradation performance was evaluated under different preparation conditions and pavement structures. The influences of void ratio, photocatalyst dosage, pavement type, and pavement thickness on the exhaust degradation function were examined. The degradation rates of NO and CO among the four monitored pollutants were observed to follow a three-stage pattern of “slow–fast–steady”, while the degradation rates of NO_2_ and HC followed a “fast–slow–steady” pattern. Increasing the void ratio and the photocatalyst dosage yielded similar effects on exhaust degradation efficacy, enhancing the degradation rate and reducing the time required to reach equilibrium. The increase in the void ratio of porous asphalt mixtures and porous cement concrete reduced the time required to reach equilibrium by an average of 4.4 and 2.3 min for the four pollutants monitored, respectively. Increasing the dosage of photocatalytic material by 2 kg/m^3^ increased NO degradation by an average of 1.5% and reduced the time required to reach equilibrium by an average of 0.8 min. The degradation rate of porous cement concrete in the first reaction stage was faster than that of porous asphalt mixtures, and the time required to reach equilibrium state increased by 2 min compared to that of porous asphalt mixture. And the impact of specimen thickness on exhaust degradation performance was minimal.

## 1. Introduction

In recent years, the density of road networks and the number of vehicles have surged significantly due to advancements in transportation infrastructure and enhancements in living standards. According to estimates by Hedges et al., there are approximately 1.446 billion cars worldwide [1], offering substantial support for road transportation and mobility of the population. Simultaneously, the developed urban road network facilitates the fundamental conditions for dispersing and alleviating traffic congestion, offering residents a greater array of travel route options [2]. However, this also results in substantial vehicle exhaust emissions. According to statistics, global vehicles release millions of tons of exhaust annually. In China alone, the total emissions of four pollutants (CO, HC, NO_X_, PM) from vehicles reached 15,577,000 tons in 2021 [3,4,5]. Vehicle emissions have emerged as a significant source of air pollution.

The intensity and dispersion patterns of vehicle exhaust emissions, along with their impact on human health, are influenced by a multitude of factors. Kuppili et al. [6] observed that operating conditions with speeds below 10 km/h resulted in a threefold increase in exhaust emissions. Due to the growth of the roadside economy, many roads are now flanked by high-rise buildings, exacerbating traffic congestion. Vehicles operating at low speeds or idling for extended periods contribute to more severe exhaust emissions [7]. The primary vehicular exhaust pollutants affecting human health are CO, HC, NO_X_, PM, and SO_2_, which are predominantly distributed at the height of human respiration [8]. Among these, CO, PM, and HC pose significant risks to the human circulatory and respiratory systems, potentially leading to conditions such as bronchitis, coronary heart disease, and heart failure. Perez et al. [9] found that 14% of asthma cases in Europe are related to vehicle exhaust emissions. In addition, NO_X_ and SO_2_ prone to combine with water vapor in the atmosphere to form acid rain, which seriously endangers the ecological environment and human health [10].

To mitigate the environmental pollution and health hazards caused by vehicle exhaust, some studies have attempted to transform traditional dense pavements into photocatalytic exhaust degradation pavements, but the primary focus of traditional dense pavement structures is on structural stability. This results in the photocatalysts within the pavement material being unable to interact with light and exhaust pollutants, thereby preventing them from fully exhibiting photocatalytic activity. Ma et al. [11] directly incorporated nano TiO_2_ into asphalt and mixed it at 170 °C to prepare modified asphalt capable of degrading NO_X_ and HC. Carneiro et al. [12] used TiO_2_ as an additive in asphalt mixtures and verified its feasibility in promoting organic degradation through methylene blue degradation tests, discovered that when the TiO_2_ content was 6% by weight of the asphalt, the photocatalytic efficiency reached a maximum of 45%. Poon and Cheung [13] prepared concrete specimens with dimensions of 200 × 100 mm and placed a 5 mm thick layer of photocatalytic concrete on top, with TiO_2_ content ranging from 0% to 10%. NO_X_ degradation tests were conducted in a closed indoor environment. The results showed that the NO_X_ degradation rate gradually decreased as the specimen age increased, ultimately reducing by approximately 8%. The sample with 10% TiO_2_ content exhibited the highest pollutant degradation rate compared to samples with lower TiO_2_ content. Sikkema et al. [14] prepared cement concrete panels containing TiO_2_ and conducted simulation tests to investigate the effects of environmental factors such as temperature, UV radiation intensity, airflow rate, and relative humidity on their photocatalytic performance. The results indicated that temperature and UV irradiance positively influenced the photocatalytic rate, while airflow rate and relative humidity inhibited the degradation process.

Meanwhile, this design also leads to issues such as water drift, spray, and damage to the underlying soil environment during rainy conditions [15]. Porous pavements utilize permeable materials, which allow photocatalytic material to be incorporated in pavement materials exposed to both light irradiation and exhaust gases. And the permeability enhances road conditions in wet weather. Additionally, this contributes to the enhancement of the urban hydrological environment. With the implementation of the “dual-carbon strategy”, there is increasing emphasis across various sectors on ecological and environmental protection benefits. Enhancing and expanding the environmental protection functions of pavement materials has become a focal point of research in related fields [16]. Incorporating exhaust degradation and water permeability into pavement design is an effective strategy to address exhaust pollution and driving safety issues on rainy days caused by dense pavements [17].

Several researchers have integrated photocatalytic materials into porous pavements to impart exhaust degradation capability to the pavement. Shen et al. [18] discovered that the open pore structure of pervious concrete can shield TiO_2_ particles from traffic loading and environmental weathering. Additionally, the pore structure of pervious concrete aids in the purification of runoff. Mousavi Rad et al. [19] prepared porous asphalt pavements doped with nano-TiO_2_ and found that the pavement effectively degraded NO_3_^⁻^ and SO_2_^⁻^ in the runoff when the nano-TiO_2_ doping level was 7%. Jimenez-Relinque et al. [20] identified the key indicators for evaluating photocatalytic performance based on experimental data. And the photocatalytic efficacy of pavements was assessed using Photoscaling Decision Maker software (https://www.life-photoscaling.eu/decisionmaker/, accessed on 11 December 2024). Zhang et al. [21] conducted a comprehensive study on photocatalytic asphalt mixtures with internal doping, determining the optimal formulation as follows: the photocatalytic material used was anatase TiO_2_, with an optimal content of 3.1%, the binder was TPS-modified asphalt, and the gradation adopted was PAC-13. Hu et al. [22] used iron-doped titanium oxide as the fine aggregate in open-graded friction course (OGFC-16) asphalt mixtures, developing a green and sustainable photocatalytic porous asphalt pavement material. Dylla et al. [23] determined the optimal porosity, thickness, and TiO_2_ content for photocatalytic permeable cement concrete based on NO_X_ degradation efficiency. The recommended values were 27% porosity, 75 mm thickness, and 3–5% TiO_2_ content. They also found that for every 25.0 mm increase in the thickness of the photocatalytic layer in the permeable cement concrete, the NO_X_ degradation efficiency increased by approximately 3.60%.

In addition, the interconnected voids of porous pavement enable the infiltration and drainage of surface water. This effectively diminishes surface runoff and enhances driving safety during rainy conditions. The majority of research on porous pavement materials concentrates on aspects such as mix design, drainage functionality, and skid resistance enhancement. Zhou et al. [24] proposed a porous cement concrete base layer with drainage capabilities and conducted a systematic study on the design of its material composition. Saudo-Fontaneda et al. [25] analyzed the advantages and feasibility of porous cement concrete as a drainage and skid-resistant pavement material, summarizing the primary challenges and solutions associated with porous pavements. Rasool et al. [26] paved roads with porous asphalt mixtures and followed up and found that porous pavement ensures rapid rainwater infiltration.

In addition to exhaust degradation pavements, the main instruments for motor vehicle exhaust control are currently focused on vehicle production and sales, road alignment optimization and internal and external purification measures for internal combustion engines. Most industrialized countries have established controls or solutions for motor vehicle exhaust pollution, with the United States being the first country to establish standards for passenger vehicle exhaust emissions. The United States established the Energy Policy and Conservation Act (EPCA) [27] in the 1970s, but maintained the standards for 25 years without updating them. In the meantime, European countries, Japan, China, and California have repeatedly updated their exhaust emission requirements for newly manufactured motor vehicles in response to advances in internal combustion engine technology and atmospheric degradation. California’s requirements for harmful emissions from motor vehicles are more stringent than those of other parts of the world. Since 2002, California legislation has required the California Air Resources Board (CARB) to reduce motor vehicle tailpipe emissions to the maximum extent economically, efficiently, and practicably possible; CARB estimates that the more stringent emission standards will result in emissions of exhaust pollutants by 17 percent in 2020 and 25 percent in 2030 [28]. However, CARB also recognizes that regulatory limits on motor vehicle emissions will be offset by growth in motor vehicle ownership and miles travelled, which will stabilize at today’s GHG emission levels by 2030. In addition, the adoption of more advanced engine technologies is another effective way to reduce exhaust pollutant emissions in spite of improving the combustion efficiency of gasoline and diesel fuel. Low-temperature combustion (LTC) technologies such as homogeneous charge compression ignition and premixed compression ignition can simultaneously reduce the concentrations of NO_X_ and PM in exhaust gases. Some studies have even shown that exhaust gas can reach the requirements of emission standards even without off-board purification after the adoption of these technologies [29].The consideration of energy saving and emission reduction in the road sector is mainly reflected in the linear design, and most of them are still only in the research stage. The level and longitudinal linear indicators of highways affect motor vehicle engine load and acceleration frequency, which will further affect motor vehicle fuel consumption and exhaust emissions [30,31,32].

Researchers generally focus on the permeability and load-bearing capacity of porous pavement materials and the enhancement of photocatalytic performance for exhaust degradation. Most of the studies on applying photocatalytic materials in porous pavement materials are compared with dense pavement materials. However, there is insufficient detail regarding the impact of factors such as the type of porous pavement material, void ratio, and paving thickness on exhaust degradation performance and permeable efficacy. In this paper, anatase-type titanium dioxide nanoparticles are used as photocatalytic materials. It is integrated into asphalt mixtures and porous concrete by replacing part of the mineral powder or gelling material, creating porous pavement materials with exhaust degradation capability. This study explores the photocatalytic reaction characteristics of different exhaust pollutants and assesses how factors such as porosity, photocatalyst dosage, pavement type, and thickness influence exhaust gas degradation efficiency. This research aims to guide the application and promotion of photocatalytic materials in porous pavement systems.

## 2. Materials

### 2.1. Photocatalytic Material

Nano titanium dioxide is the most widely used photocatalytic material in pavement applications due to its uniform particle size, large specific surface area, excellent dispersion, strong nano effect, and stable photocatalytic performance [33]. The structure of anatase TiO_2_ crystals features layer dislocations and displacements, which facilitate the generation and separation of charge carriers, resulting in high catalytic activity of the generated carriers. Therefore, in this study, anatase nano-TiO_2_ produced by Sinopharm Chemical Reagent Co., Ltd. was used as the photocatalytic material, with its technical specifications detailed in Table 1.

### 2.2. Photocatalytic Porous Asphalt Mixtures

**Figure 1:**
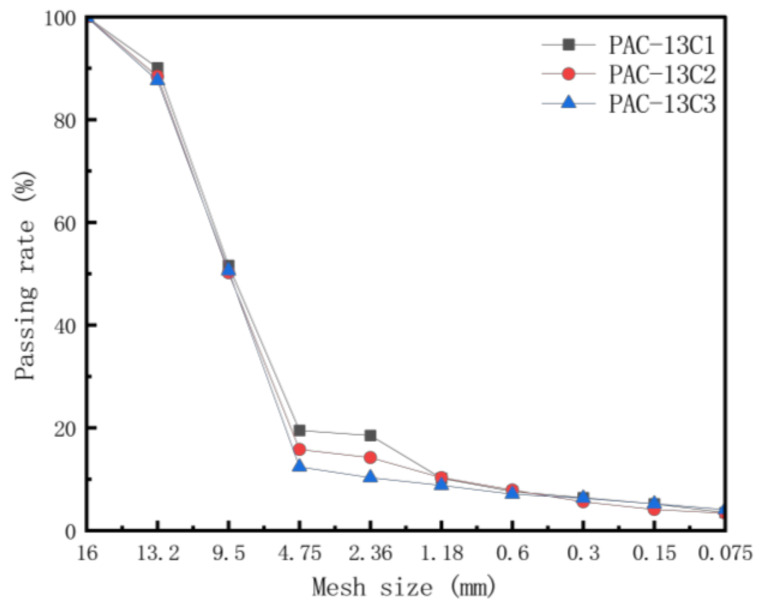
Internal pore distribution in porous materials.

The proportion design of the porous asphalt mixture was conducted according to the reference gradation provided in the “Technical Specification for Permeable Asphalt Pavement” (CJJ/T 190-2012) [34]. The gradation for the porous asphalt mixture is depicted in Figure 1. According to the “Standard Test Methods of Bitumen and Bituminous Mixtures for Highway Engineering” (JTG E20-2011) [35], the optimal asphalt content for PAC-13C1, PAC-13C2, and PAC-13C3 is 4.90%, 4.30%, and 4.40%, respectively. Based on the connected void rate test, the void ratios of PAC-13C1, PAC-13C2, and PAC-13C3 are 18%, 21%, and 24%, respectively [36]. Nano titanium dioxide was incorporated into the porous asphalt mixtures by partially replacing the mineral powder. The detailed parameters of the exhaust degradation porous mixtures prepared in this study with varying photocatalyst content and gradation are presented in Table 2.

To investigate the influence of pavement thickness on exhaust degradation efficiency and to provide a basis for optimizing the structure of exhaust degradation porous pavements, slab specimens of varying thicknesses were molded using a self-designed preparation method. The exhaust degradation efficacy of these specimens was tested under a consistent photocatalyst dosage of 6.0 kg/m^3^. The specimens required for the test are detailed in Table 3.

Photocatalytic porous asphalt mixtures were produced according to the following procedure. (1) Dry the aggregate at 180 °C until constant weight. Heat the asphalt at 160 °C to a liquid with fine flowability. (2) Add the aggregate into the asphalt mixture mixer at 160 °C and mix for 40 s. (3) Add the asphalt into the asphalt mixture mixer and mix for 90 s. (4) Add the mineral powder and photocatalytic materials, mixing 50 s to obtain the photocatalytic porous asphalt mixtures.

Given that the thickness of the slab specimen in JTG E20-2011 is 5 cm and most existing molds range from 5 to 10 cm, the following technical solution is proposed to prepare plate specimens of varying thicknesses. (1) Considering the temperature conditions during the molding process, nylon pads of different thicknesses were used to prepare specimens with a thickness of less than 5 cm. (2) The quantities of aggregate, asphalt, and other materials used in molding plate specimens of different thicknesses were adjusted proportionally based on the specimen’s volume. (3) The standard plate specimen mold should be thoroughly cleaned before molding the specimen. When placing the pad into the mold, the pad should be in close contact with the bottom of the steel mold. (4) The specimen should be prepared according to the requirements specified in T0703 of JTG E20-2011 [35].

### 2.3. Photocatalytic Porous Cement Concrete

In this study, the proportion design of porous cement concrete was conducted using the volumetric method. The results of the proportion design and porous cement concrete gradation are presented in Table 4 and Table 5. The test conditions for varying photocatalytic material dosage and gradations are detailed in Table 6.

The production of photocatalytic porous cement concrete refers to the following process. Firstly, mix the aggregate and 50% water for 25~30 s. Next, add the cementitious material, admixture and photocatalytic materials, then mix for 35~40 s. Finally, add the remaining water and mix for more than 50 s to obtain the photocatalytic porous cement concrete.

## 3. Test Methods

**Figure 2:**
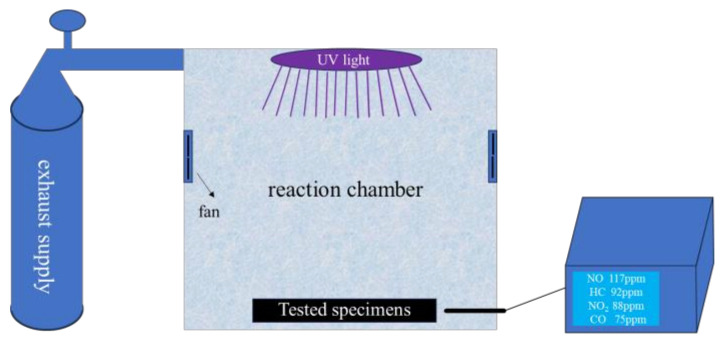
Actual effect diagram of exhaust degradation simulation device.

Given the lack of standardized test equipment for evaluating exhaust degradation efficiency of pavement materials, this study developed a test device for measuring exhaust degradation efficiency, as illustrated in Figure 2. The device comprises an exhaust supply unit, an exhaust degradation simulation chamber, and a real-time exhaust concentration monitoring system [37,38]. The gas components and their ratios in the exhaust supply device are detailed in Table 7. Additionally, based on existing calculation and evaluation methods for photocatalytic exhaust degradation and considering current technical characteristics, the degradation rate (k), the average degradation rate (V), and the 30 min degradation rate (J_30min_) were selected as the evaluation indices [17].

**Table 7:** Gas types and proportion inside the exhaust gas supply device.

Types	C_3_H_8_	CO	CO_2_	NO	N_2_
Proportion/%	6	5	12	25	52

(1) $$V=\frac{c_{\max}-c_{\min}}{T}$$

where *V* is the average degradation rate, *T* is the degradation reaction time, *c_max_* is the maximum value of the initial gas concentration, and *c_min_* is the minimum value of the gas concentration.

(2) $$J_{30\min}=\frac{n_{0}-n_{30\min}}{n_{0}}$$

where *n*_0_ is the initial gas concentration and *n*_30min_ is the gas concentration 30 min after the start of the test. The exhaust gas degradation performance test is conducted following these steps. After completing the commissioning and preheating of the exhaust gas testing equipment, the slab specimens are placed into the test chamber. The exhaust is then introduced until the concentration reaches the predetermined value. The UV lamp is then turned on to begin the test. NO, NO_2_, CO, and HC concentrations are recorded every minute until no significant changes are observed in the data.

**Figure 3:**
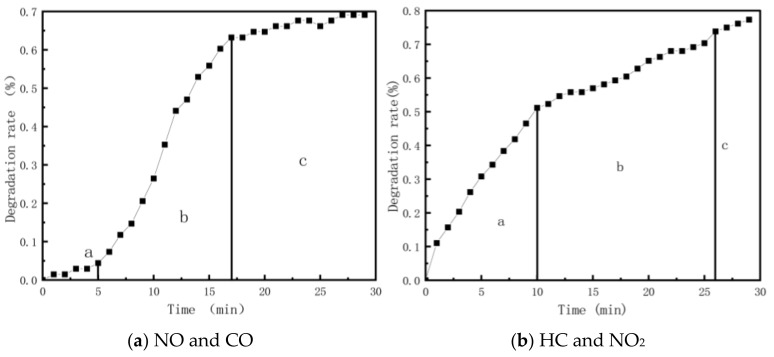
Schematic diagram of three stages of exhaust gas degradation.

Based on the photocatalytic reaction rate characteristics of different exhaust components, a “three-stage” model is proposed, as illustrated in Figure 3. The three-stage reaction rate for NO and CO follows a “slow–fast–stable” pattern, while for HC and NO_2_, it follows a “fast–slow–stable” pattern. In the three-stage model, the critical times “a–b” and “b–c” are designated as P and Q, respectively. These times can be utilized to assess the reaction state and evaluate the photocatalytic reaction under different conditions.

## 4. Results and Discussions

### 4.1. Photocatalytic Reaction Characteristics of Different Exhaust Pollutants

**Figure 4:**
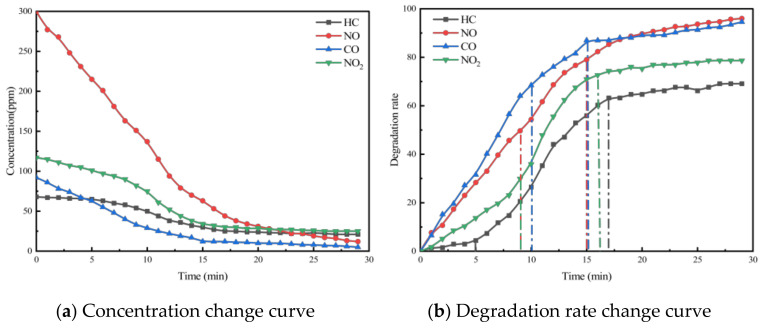
Exhaust gas degradation curve of PA-7C2T5.

Taking PA-7C2T5 in Table 2 as an example, the test was conducted according to the experimental method in Section 3. And the concentration change curves of different pollutants were recorded, as shown in Figure 4a. At the same time, to analyze the relative concentration changes in pollutants more intuitively, the degradation rate curves of different pollutants were also plotted, as shown in Figure 4b.

The concentration and degradation rate curves for the four pollutants (HC, NO, CO, and NO_2_) within the reaction chamber of the PA-7C2T5 during the exhaust degradation efficacy test are illustrated in Figure 4.

It was observed that the concentrations of all four exhaust pollutants monitored in the reaction chamber during the test exhibited a decreasing trend over time. There are two reasons for the gradual decline in the concentrations of the four components. Firstly, under ultraviolet light irradiation, nano titanium dioxide possesses a strong redox capacity, which leads to the decomposition of nitrogen oxides and hydrocarbons into nitrate, water, and carbon dioxide, respectively. Secondly, CO and NO are easily converted to other substances due to their chemical instability.

Additionally, as shown in the figure, the concentration change patterns of the various exhaust components exhibit a trend where the rate of decrease is initially rapid and then gradually slows down. The rate of chemical reactions is influenced by factors such as the concentration of reactants, temperature, pressure, and the presence of a catalyst. In this experiment, environmental conditions like temperature were controlled using a self-developed experimental setup, ensuring that the reaction rate was primarily dependent on reactant concentration and chemical activity. As the reaction progresses, the pollutants in the reaction chamber are continuously consumed, causing the concentration to gradually decrease. This reduction in concentration leads to a lower probability of effective intermolecular collisions.

On the other hand, due to the varying initial concentrations and reactivities of the four monitored exhaust pollutants, the patterns of change exhibited distinct differences. According to Table 7 and Figure 4, the initial concentration of each pollutant varies, with the concentration order being NO, NO_2_, CO, and HC. In the first 5 min of the test, the reaction rate of NO is significantly higher than that of the other pollutants. This is due to NO’s concentration advantage, which results in a much higher probability of contact with the redox sites on the surface of the photocatalytic material compared to the other pollutants. Subsequently, the degradation rates of NO and CO gradually begin to decrease, while the rate of decrease in the concentration of HC and NO_2_ starts to increase. This is because the degradation reaction alters the ratio of pollutant concentrations in the reaction chamber, leading to a relative increase in the concentrations of HC and NO_2_ compared to the other two pollutants. Additionally, NO_2_ is an intermediate product in the degradation of NO. Therefore, despite its higher concentration, the degradation rate of NO_2_ is lower than NO. For PA-7C2T5, the concentrations of all monitored contaminants essentially stabilized 17 min after the start of the test. In other test conditions, the patterns of change in monitored pollutant concentrations were generally similar.

### 4.2. Influence of Porous Mixture Parameters on Exhaust Degradation Efficiency

#### 4.2.1. Void Ratio

1. Porous asphalt mixtures

**Figure 5:**
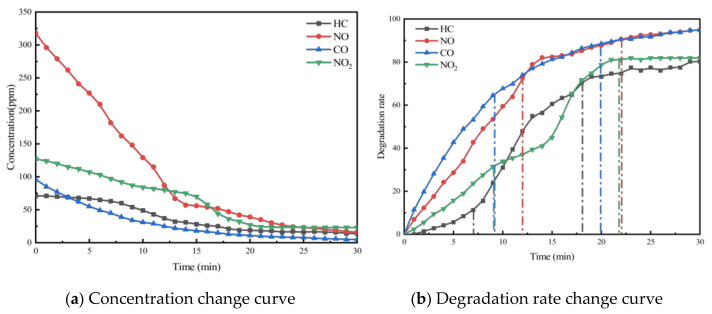
Exhaust gas degradation curve of PA-6C1T5.

**Figure 6:**
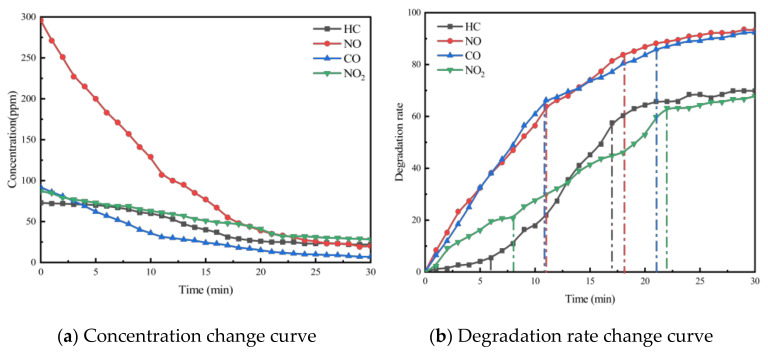
Exhaust gas degradation curve of PA-6C2T5.

**Figure 7:**
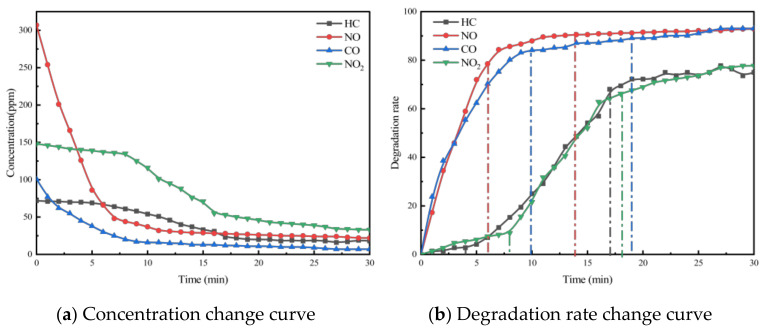
Exhaust gas degradation curve of PA-6C3T5.

To investigate the effect of void ratio on the exhaust degradation efficacy of porous asphalt materials, tests were conducted following the specimen preparation conditions for PA-6C1T5, PA-6C2T5, and PA-6C3T5, with void ratios of 18%, 21%, and 24%, respectively, as outlined in Table 2. The results of these experiments are presented in Figure 5, Figure 6 and Figure 7.

From the figures, it can be observed that the concentration trends of the four exhaust components in the exhaust degradation efficiency tests of specimens with different void ratios are generally similar. All the pollutants exhibit a decreasing trend over time, and this decline follows the pattern noted in Section 4.1.

The relationship between degradation efficiency and the void ratio was analyzed, revealing that a larger void ratio results in higher photocatalytic reaction efficiency. In the exhaust degradation efficiency test, the P and Q points of the reaction rate curves for specimens with larger void ratios were advanced to varying degrees. The comparison of data from each test group shows that under the same photocatalytic material dosage, specimens with larger void ratios reach the relative equilibrium reaction state earlier. This indicates that increasing the void ratio accelerates the rate of the photocatalytic reaction. The contact between photocatalytic materials and reactants, as well as the absorption of light radiation, are both essential conditions for the photocatalytic reaction. Consequently, photocatalytic materials that are entirely coated by asphalt mastic cannot participate in the photocatalytic reaction. The larger void ratio increases the specific surface area of the porous asphalt mixture specimen, exposing more asphalt slurry to air and light. This exposure allows more photocatalytic material to participate in the photocatalytic reaction. From a microscopic point of view, the increase in the void ratio allows more photocatalytic materials to be exposed to the exhaust pollutant environment, enabling them to absorb light at the desired wavelength. As a result, more TiO_2_ reaches its activated state, increasing the likelihood of effective collisions between reactants and accelerating the photocatalytic reaction.

2. Porous cement concrete

**Figure 8:**
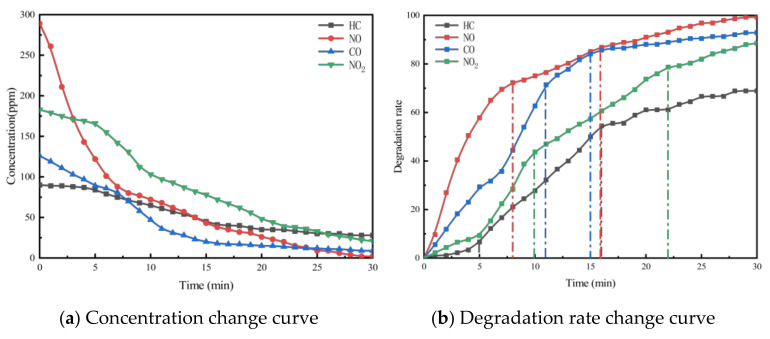
Exhaust gas degradation curve of PC-613T5.

**Figure 9:**
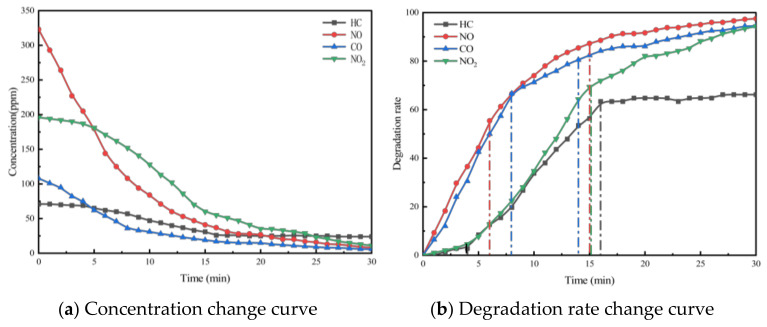
Exhaust gas degradation curve of PC-616T5.

To investigate the effect of void ratio on the photocatalytic efficacy of porous cement concrete, photocatalytic exhaust degradation tests were conducted on PC-613T5 and PC-616T5, as listed in Table 3, respectively. These tests were performed under a photocatalytic material dosage of 6 kg/m^3^. The results are presented in Figure 8 and Figure 9.

As seen in Figure 8 and Figure 9, the same as porous asphalt mixtures, the increase in void ratio for porous cement concrete does not affect the final reaction equilibrium state. However, the required time for the exhaust pollutant concentration to reach the equilibrium state decreases with the increase in the void ratio. Therefore, the increase in the void ratio favors the photocatalytic reaction.

#### 4.2.2. Photocatalyst Dosage

**Figure 10:**
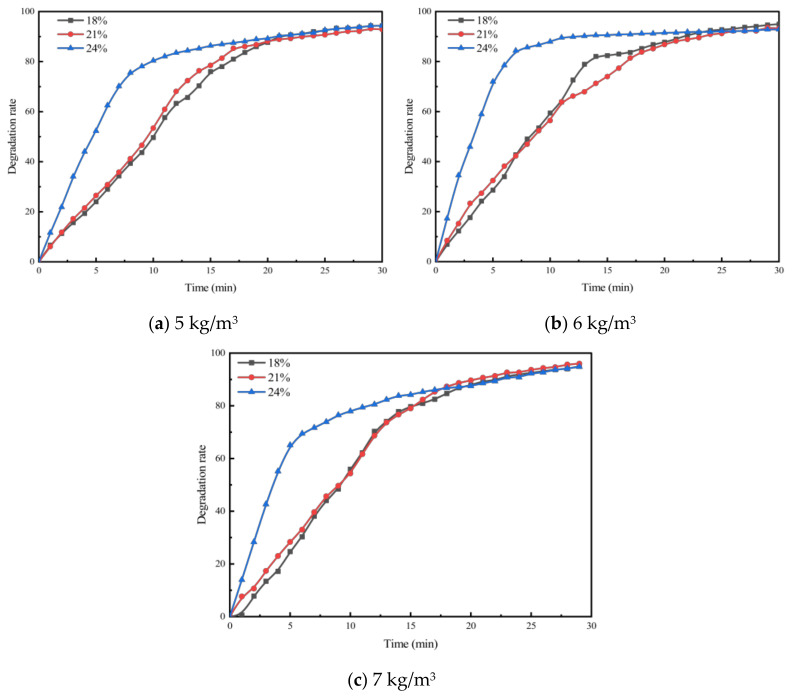
NO degradation efficiency of specimens with different photocatalyst dosages.

To investigate the effect of photocatalyst dosage on the exhaust degradation efficiency of porous mixtures, asphalt mixture specimens with different photocatalyst dosages were tested according to the conditions in Table 2 and the methodology in Section 3. NO was used as the characteristic component to examine the impact of photocatalyst dosage on degradation efficiency, and the results are shown in Figure 10.

From Figure 10, it can be observed that the trends in NO degradation rate under different photocatalyst dosages are essentially the same. As the photocatalyst dosage increases, the degradation rate curve shifts upward and reaches the P and Q points earlier, indicating that the reaction equilibrium is attained more quickly. This suggests that increasing the photocatalyst dosage promotes the photocatalytic reaction, similar to how the reaction rate is enhanced by increasing the void ratio. Both factors contribute to accelerating the photocatalytic process. The increase in photocatalyst dosage raises the redox potential exposed to the exhaust environment, thereby increasing the likelihood of effective molecular collisions and enhancing the photocatalytic reaction rate. In addition, the increase in photocatalyst dosage and void ratio have similar effects on the photocatalytic exhaust degradation efficiency. Neither can alter the final equilibrium state of the exhaust pollutants. However, both increase the reaction rate and shorten the time required to reach equilibrium.

#### 4.2.3. Coupled Effects of Void Ratio and Photocatalytic Material on Degradation Efficiency

**Figure 11:**
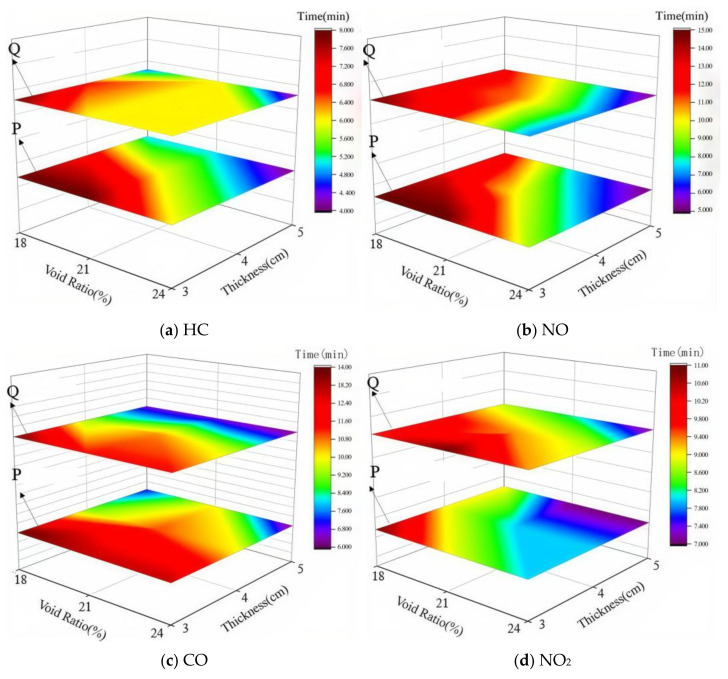
Trends in P and Q points under different conditions.

To investigate the variation in exhaust degradation efficacy of porous mixtures under the combined influence of void ratio and photocatalyst dosage, tests were conducted on various specimens following the conditions in Table 2 and the methodology outlined in Section 3. This section explores the coupled effect of void ratio and photocatalyst dosage by analyzing changes in the three-stage critical points of the exhaust degradation rate under different testing conditions. The results are shown in Figure 11.

In Figure 11, the lower layer depicts point P, while the upper layer represents point Q. The *X*-axis corresponds to the void ratio, and the *Y*-axis reflects the dosage of photocatalytic material. Different colors are used to illustrate the changes in the critical points P and Q. From the analysis results of Section 4.2.1 and Section 4.2.2, it can be observed that the critical points of P and Q are advanced to varying degrees as both the void ratio and the amount of photocatalytic materials increase. From Figure 11, it can be seen that the P and Q points reach their minimum values at the sharp corners on the right side of the diagram. This suggests that the increase in void ratio and photocatalytic material dosage has a synergistic effect in enhancing the photocatalytic reaction.

### 4.3. Influence of Pavement Structure on the Efficiency of Exhaust Degradation

#### 4.3.1. Pavement Materials

**Figure 12:**
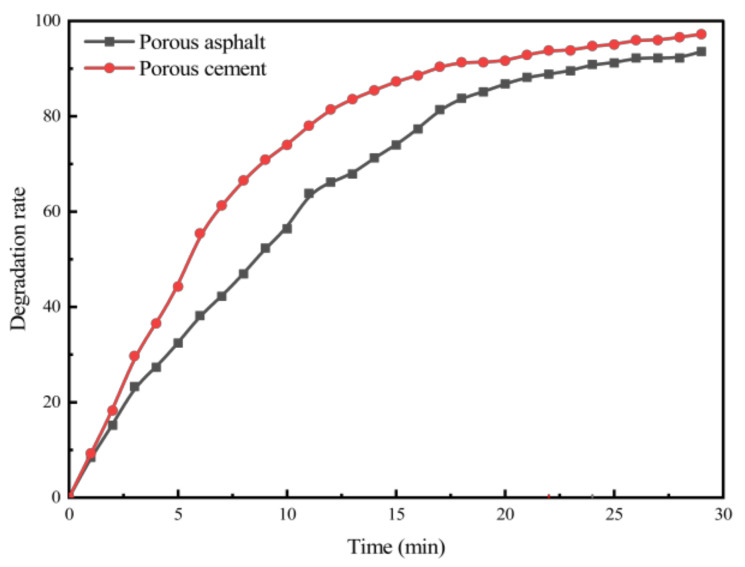
NO degradation rate of asphalt and cement specimens with the same porosity and photocatalyst content.

In this section, to investigate the differences in exhaust degradation efficiency between porous cement concrete and asphalt mixtures, specimens with the same void ratio and photocatalytic dosage were subjected to exhaust degradation efficacy tests. NO was used as the characteristic component to analyze the exhaust degradation efficacy of the two groups. The test results are presented in Figure 12.

As seen in Figure 12, the NO degradation rate curves of different material types, under identical void ratio and photocatalyst dosage conditions, exhibit a high degree of similarity in terms of NO degradation efficiency. From the curve of different types of permeable pavement can be seen that the degradation rate of permeable cement concrete in the pre-reaction period is faster than that of permeable asphalt mixture, and the time to reach the relative equilibrium state of the reaction is shorter. This may be because permeable cement concrete is a white pavement, which is more favorable for the photocatalytic material to receive UV and visible light. On the other hand, cement is not as effective as asphalt in coating the photocatalytic material nanoparticles, which leaves more photocatalytic material nanoparticles exposed to the air, resulting in a faster reaction rate.

#### 4.3.2. Influence of Pavement Thickness on Exhaust Degradation

**Figure 13:**
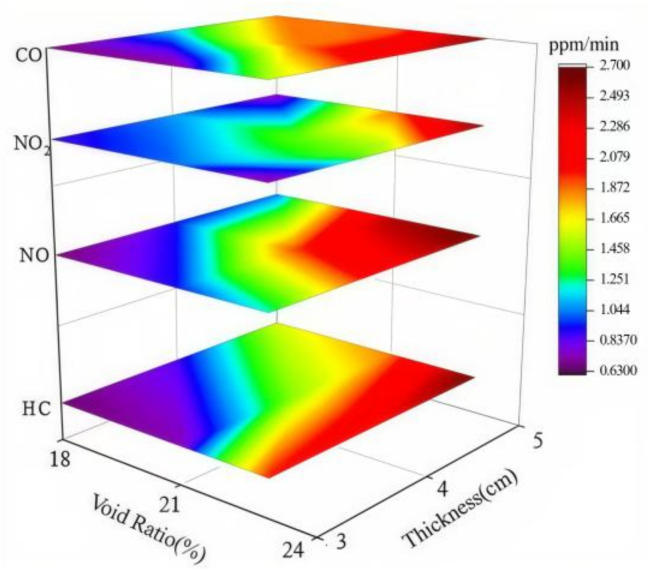
Average degradation rate of plate specimens of different thicknesses.

**Figure 14:**
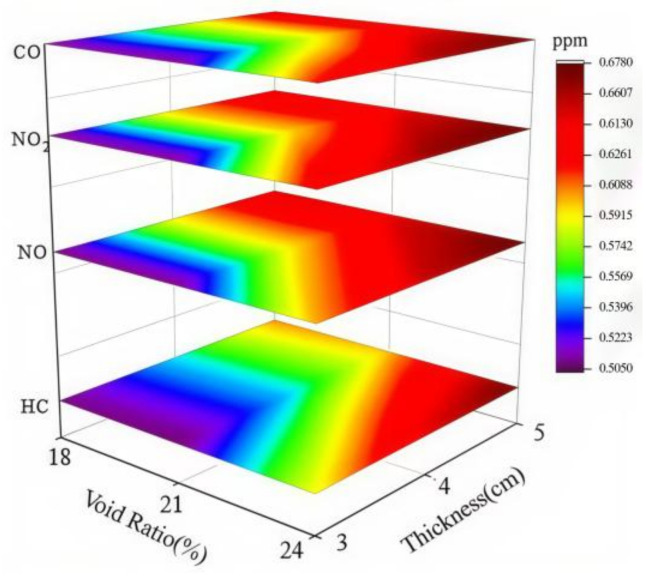
30 min degradation rate of plate specimens with different thicknesses.

According to the test conditions in Table 3, the effects of pavement thickness on exhaust degradation efficiency were studied. The average degradation rate and the 30 min degradation rate were calculated. The test results are shown in Figure 13 and Figure 14.

Figure 13 and Figure 14 show that pavement thickness has minimal impact on exhaust degradation efficiency at different void rates. As specimen thickness increases, both the average degradation rate and the 30 min degradation rate improve. This improvement is more significant with a larger void ratio, where the NO degradation rates of PAC-C1, PAC-C2, and PAC-C3 increased by 0.5%, 1.3%, and 1.8%, respectively. This suggests that the effective depth of specimens with a larger void ratio increases more within the same thickness variation range. The reason is that the beneficial effect of increased thickness is more pronounced in specimens with larger void ratios, as they allow greater light penetration and stronger radiation intensity within the voids. Considering porous pavement as functional pavement, although light and exhaust components can enter the voids and initiate photocatalytic reactions, the penetration depth of light and the diffusion of the exhaust is limited by thickness. Thus, excessive structural thickness offers limited functional benefits. Therefore, it is recommended that the material be applied only in the upper layer, with a recommended pavement thickness range of 3 to 5 cm.

### 4.4. Optimal Materials and Structures for Photocatalytic Pavements

The effects of photocatalytic material dosage, void ratio, material type and functional layer thickness on the exhaust degradation performance of photocatalytic pavement were explored in the previous section, and based on the above experimental results, the optimal combination of materials and structures for photocatalytic pavement was determined as shown in Table 8.

## 5. Conclusions

In this paper, the exhaust degradation efficacy of porous mixtures was tested under varying preparation conditions and pavement structures using a self-developed exhaust degradation efficacy test device and method. The effects of void ratio, photocatalytic material dosage, pavement type, and pavement thickness on the exhaust degradation function were examined. The main conclusions are as follows.

1. In the exhaust degradation test, the concentrations of different exhaust components exhibited a decreasing trend. However, the photocatalytic reaction rates of the various exhaust components differed. The reaction rates of NO and CO followed the “slow–fast–steady” model, while the reaction rates of NO_2_ and HC followed the “fast–slow–steady” model. This paper proposes using two critical points, P and Q, from these three stages to evaluate the photocatalytic reaction characteristics.
2. The increase in the void ratio does not affect the final equilibrium state of the photocatalytic reaction. However, whether in the case of porous asphalt mixture or porous 435 cement concrete, as the void ratio increases, the time required for the photocatalytic reaction to reach equilibrium decreases, and the reaction rate accelerates. The increase in the void ratio of porous asphalt mixtures and porous cement concrete reduced the time required to reach equilibrium by an average of 4.4 and 2.3 min for the four pollutants monitored, respectively.
3. The effect of increasing photocatalyst dosage on photocatalytic exhaust degradation efficiency is similar to that of increasing the void ratio. While it does not alter the final equilibrium state of the reaction, it enhances the reaction rate and shortens the time required to reach equilibrium. Increasing the dosage of photocatalytic material by 2 kg/m^3^ increased NO degradation by an average of 1.5% and reduced the time required to reach equilibrium by an average of 0.8 min.
4. The NO degradation rate curves of porous mixtures of different material types under the same conditions exhibit high similarity. However, the degradation rate of cement concrete in the initial reaction phase is faster than that of porous asphalt mixture, and the reaction time to reach relative equilibrium state was 2 min earlier than that of porous asphalt mixtures.
5. As the specimen thickness increases, both the average degradation rate and the 30 min degradation rate improve. However, due to the limited penetration depth of light radiation and exhaust diffusion, a greater thickness has limited significance for the exhaust degradation performance of the pavement.
6. This study has not yet evaluated the functional durability of exhaust degradation pavements, or the comprehensive economic and environmental benefits of exhaust degradation pavements. Meanwhile, the difficulties faced in the current research on exhaust degradation pavements are the functional evaluation of exhaust degradation pavements in real road environments and the measurement of cumulative degradation pollutants. These directions should be paid attention to in further research.

## Figures and Tables

**Table 1:** Technical indicators of nano TiO_2_.

Appearance	pH	Crystalline Phases	Purity (%)	Particle Diameter (nm)	BET (m^2^/g)	Surface Modifications
White powder	6–8	Anatase	>99.9	18	150–200	unmodified

**Table 2:** The dosage of photocatalytic materials in porous asphalt mixtures.

Number	Types of Photocatalytic Materials	Photocatalytic Material Dosage (kg/m^3^)	Gradation Types	Thickness (cm)
PA-0C1T5	nano TiO_2_	0	PAC-13C1	5
PA-0C2T5	nano TiO_2_	0	PAC-13C2	5
PA-0C3T5	nano TiO_2_	0	PAC-13C3	5
PA-5C1T5	nano TiO_2_	5.0	PAC-13C1	5
PA-5C2T5	nano TiO_2_	5.0	PAC-13C2	5
PA-5C3T5	nano TiO_2_	5.0	PAC-13C3	5
PA-6C1T5	nano TiO_2_	6.0	PAC-13C1	5
PA-6C2T5	nano TiO_2_	6.0	PAC-13C2	5
PA-6C3T5	nano TiO_2_	6.0	PAC-13C3	5
PA-7C1T5	nano TiO_2_	7.0	PAC-13C1	5
PA-7C2T5	nano TiO_2_	7.0	PAC-13C2	5
PA-7C3T5	nano TiO_2_	7.0	PAC-13C3	5

**Table 3:** Gradation and thickness of test specimens.

Number	Gradation	Specimen Thickness (cm)
1	PAC13-C1	5
2	PAC13-C1	4
3	PAC13-C1	3
4	PAC13-C2	5
5	PAC13-C2	4
6	PAC13-C2	3
7	PAC13-C3	5
8	PAC13-C3	4
9	PAC13-C3	3

**Table 4:** Porous cement concrete proportions.

Void Ratio (%)	C/A	Cement (kg/m^3^)	Water (kg/m^3^)	Coarse Aggregate (kg/m^3^)	TiO_2_ (%)
23	1:5	341.3	109.2	1706.4	0.3
25	1:6	284.4	91.0		6.9

**Table 5:** Porous cement concrete gradation.

Sieve Hole Size (mm)	Mass Percentage Through the Sieve Hole (%)	
	PC-16G	PC-13G
26.5	0	0
16	20	0
9.5	20	25
4.75	60	75

**Table 6:** The dosage of photocatalytic materials in porous cement concrete.

Number	Types of Photocatalytic Materials	Photocatalytic Material Dosage (kg/m^3^)	Gradation Types	Thickness (cm)
PC-013T5	nano TiO_2_	0	PC-13G	5
PC-016T5	nano TiO_2_	0	PC-16G	5
PC-513T5	nano TiO_2_	5.0	PC-13G	5
PC-516T5	nano TiO_2_	5.0	PC-16G	5
PC-613T5	nano TiO_2_	6.0	PC-13G	5
PC-616T5	nano TiO_2_	6.0	PC-16G	5
PC-713T5	nano TiO_2_	7.0	PC-13G	5
PC-716T5	nano TiO_2_	7.0	PC-16G	5

**Table 8:** Optimal materials and structures for photocatalytic pavements.

Material Type	Photocatalytic Material Dosage (kg/m^3^)	Void Ratio (%)	Thickness (cm)
Porous asphalt mixtures	7	24	3–5 cm
porous cement concrete	7	25	

## Data Availability

The data presented in this study are available on request from the corresponding author due to privacy.

## Abbreviations

The following abbreviations are used in this manuscript:

PA	Porous asphalt
PAC	Porous asphalt concrete
PC	Porous concrete
BET	BET surface area
P	The critical time “a–b”
Q	The critical time “b–c”
